# Relationship between Temporomandibular Disorders and Psychological and Sleep Aspects in University Teaching Staff: A Regression Model

**DOI:** 10.3390/jcm9123960

**Published:** 2020-12-07

**Authors:** Guadalupe Molina-Torres, Pablo Roman, Andrada Butilca, Nuria Sánchez-Labraca, Diana Cardona, Manuel Gonzalez-Sanchez

**Affiliations:** 1 Department of Nursing Science, Physiotherapy and Medicine, University of Almeria, 04120 Almeria, Spain; guada.lupe@ual.es (G.M.-T.); msl397@ual.es (N.S.-L.); dcardona@ual.es (D.C.); 2 Department of Nursing Science, Health Sciences Research Center, University of Almeria, 04120 Almeria, Spain; 3 Faculty of Health Sciences, University of Almeria, 04120 Almeria, Spain; andradabutilca@gmail.com; 4 Department of Physiotherapy, University of Malaga, 29071 Malaga, Spain; mgsa23@uma.es

**Keywords:** temporomandibular disorders, burnout, university, university staff, anxiety, depression

## Abstract

Aim: The objective was to analyze burnout syndrome, anxiety, depression and sleep quality in teaching and research staff in the university setting and its impact on temporomandibular dysfunction (TMD), and to analyze the psycho-emotional variables that could explain the possibility of someone suffering from TMD. Methods: A transversal study was carried out with a sample consisting of 173 participants belonging to university teaching and research staff. The correlation between variables was performed using the Pearson’s correlation coefficient. Through a linear regression, an estimate of the degree of contribution was calculated that each independent variable (burnout syndrome, anxiety, depression and sleep quality) has on the dependent variable (TMD). Results: the scores are higher in the group non-tenured staff compared to tenured staff in relation to psycho-emotional variables and TMD and how psycho-emotional variables can influence the presence or absence of temporomandibular dysfunction based on job stability, this value being higher in the group of non-tenured staff (77.8%) compared to the tenured staff (44.2%). Conclusions: The non-tenured university teaching staff demonstrate higher levels of depression, anxiety, emotional exhaustion, depersonalization and poorer sleep quality. Furthermore, these variables show a higher incidence in the probability that university teaching and/or research personnel suffer from TMD.

## 1. Introduction

Those workers who are systematically exposed to the management of people or services tend to report a higher incidence of psychological disorders that can degenerate into emotional fatigue and work exhaustion [1,2]. Some of the main consequences that can be found in people suffering from work exhaustion include low or deterioration of staff morale, stress, anxiety, psychosomatic disorders, sleep disturbances, and poor organizational commitment [3].

Previous studies have analyzed some psychological effects in professionals with a high level of personal exposure such as schoolteachers, social workers, and healthcare professionals [4]. In addition, in some cases, in addition to the professional profile that some jobs have, the chances of suffering psychological disorders are increased due to factors such as clinical care pressure, job instability, the reduction in the ratio of professionals with respect to the number of people who must attend, and the academic pressure due to excessive competitiveness [5].

Teaching at university could be a stressful profession, and many teachers are exposed to high levels of emotional distress in the workplace [6], and a series of studies have suggested that many teachers suffer from job dissatisfaction [7,8]. In higher education, there are several main causes of stress in the workplace including funding cuts, excessive work hours, lack of time and resources to respond to the demands of this conflict and role ambiguity, lack of autonomy, few opportunities for professional emancipation, low wages, and the job instability, which a significant number of teachers have [2,9]. Likewise, psychological factors could exacerbate temporomandibular disorders (TMD) [10]. Psycho-emotional factors such as stress, increase the muscle tone of the masticatory organ, including the strongest muscles of the system: the temporal and masseter muscles. Increased muscle tension becomes the source of pain symptoms of TMD [11]. There are many documents that relate stress to temporomandibular dysfunction, but no document specifically analyzes the correlation between the risk factors of suffering from TMD in people who suffer from anxiety, depression, burnout syndrome, and poor sleep quality. For this reason, the objective of this study was to analyze some of the main psychological disorders that affect university teaching staff, such as state of anxiety, burnout syndrome, depression, and sleep quality and how these psycho-emotional variables may be the cause of university teaching staff suffering from TMD. Furthermore, another objective pursued by this study is to carry out a deeper analysis of this relationship (TMD psychological disorders) considering the contractual status (contractual stability or no contractual stability) of the teaching university staff. The starting hypothesis will be that university teaching staff with contractual stability will be less prone to suffer from temporomandibular alterations as a consequence of the interaction of psychological aspects and quality of sleep compared to staff with no contractual stability.

## 2. Methods

### 2.1. Design and Participants

**Figure 1:**
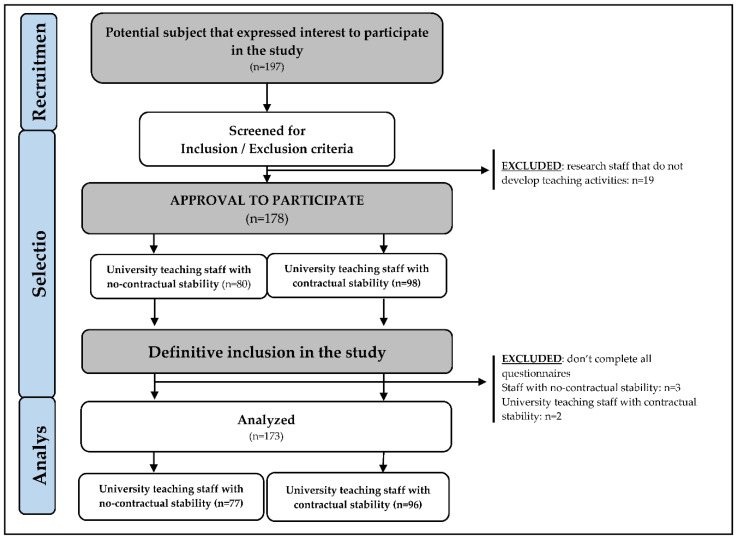
Flowchart of recruitment, selection, and analysis of participants.

A transversal study was carried out, and the participants were selected following the inclusion criteria: (1) teaching university staff from 5 different Spanish universities; and as exclusion criteria, the following were considered: (1) research staff in training that do not develop teaching activities, (2) leaving any questions unanswered on the questionnaires included in the study. The process of recruitment, selection, and final number of analyzed participants is summarized in Figure 1.

### 2.2. Ethical Considerations

The study was performed in accordance with the ethical criteria defined in the Helsinki Declaration (2013) where the ethical principles for research on human beings are defined, from the development of a national legislation for research projects (Law 223/2004 of 6 February) and confidentiality of study subjects (Law 15/1999, of 13 December). The Ethics Committee of Faculty of Health Sciences of Spain approved the completion of this study. All participants were informed about the aim of the study prior to participate. Teaching university staff participation was requested by localized email after conducting a search in the different directories of the different universities and was carried out by blind collaborators external to the study. Participants were informed about the confidentiality of their data, and all consent forms were signed.

### 2.3. Study Variables and Data Collection

Sociodemographic variables of sex, age, contractual figure, and marital status were collected as well as burnout syndrome, anxiety level, state of depression, sleep quality, and temporomandibular dysfunction. The sample was made up of 173 participants belonging to the university’s teaching staff, of which the average age was 46.78 (±8.97) years. The minimum age was 26 years, and the maximum value was 65 years, with a mean age of 41.34 (±9.36) for university teaching staff with no contractual stability and 51.15 (±5.72) for university teaching staff with contractual stability. The distribution of the sex of the participants was 46.8% female and 53.2% male. Regarding the marital status of the participants, the most prevalent status was married with 56.6%, single 22%, divorced 9.8%, widowed 1.2%, civil union 5.8%, and other 4.6%. Regarding the contractual figure at the university, university teaching staff with contractual stability positions were dominant with 55.5%, of which 41.7% are women and 58.3% are men, being 44.5% of the total sample that represents university teaching staff with no contractual stability at the university, of which 53.2% are women and 46.8% are men.

### 2.4. Measurements

In addition, to evaluate both university teaching staff with contractual stability and university teaching staff with no contractual stability in the university environment, different variables were analyzed such as the state of anxiety, depression, and quality of sleep and, on the other hand, the possibility of suffering from temporomandibular dysfunction using the Fonseca Anamnestic Index; we were thus able to establish relationships between them.

Maslach Burnout Inventory (MBI): One of the most used instruments to evaluate burnout syndrome. This scale includes 22 items and contains three subscales: a 9-item emotional exhaustion subscale with answers ranging from 5 to 45 points; a 5-item depersonalization subscale with answers ranging from 5 to 25 points; and an 8-item personal achievement in the workplace subscale with answers ranging from 5 to 40 points. The answers to the items are evaluated with a 5-point Likert-type scale that ranges from (1) never, (2) a few times a year, (3) a few times a month, (4) a few times a year, and (5) daily. High scores in the first two subscales correspond to strong feelings of burnout, while low scores in the last subscale of personal achievement in the workplace correspond to strong feelings of burnout [12]. To measure internal consistency, that is, the stability of the measure over time (a subject who responds in the same conditions to the same question, should offer the same answer), the Spanish versions of the questionnaire calculated Cronbach’s α, these values are usually stratified as follows: excellent: > 0.80; good 0.80 > reliability > 0.60; moderate: 0.60 > reliability > 0.40; poor < 0.40). The value, in the different subscales, was the following: professional achievement: Cronbach’s α = 0.85; emotional exhaustion: Cronbach’s α = 0.83; depersonalization: Cronbach’s α = 0.74 [13].

Fonseca Anamnestic Index (FAI): The presence of TMD was established with the FAI, which consists of 10 questions related to jaw movement difficulties, orofacial pain, TMJ sounds, parafunctional habits, perception of malocclusion, and emotional stress [14]. The 10 questions must be answered with “yes” (10 points), “no” (0 points), or “sometimes” (5 points), and only one answer should be marked for each question, according to its severity. The scale range is from 0 to 100 points, with high scores being very severe [15]. The sensitivity of the Fonseca Anamnestic Index in the diagnosis of temporomandibular disorders was 96%, which is an adequate value for the validation of a diagnostic test, and its specificity was 95%, also being an adequate value for the validation of a diagnostic test [16].

Beck Depression Inventory (BDI): A 21-item self-report instrument designed to assess the severity of depressive symptoms in adults and adolescents from a minimum age of 13 years. In each item, the person has to choose from a set of four alternatives, ordered from least to most serious and the phrase that best describes their condition during the last two weeks, including the day they complete the instrument. As soon as it is corrected, each item is valued from 0 to 3 points depending on the chosen alternative, then directly added to the score of each item. The total score varies from 0 to 63 [17,18]. The internal consistency of the Spanish version has a Cronbach’s α = 0.89 [19].

State and Trait Anxiety Questionnaire (STAI): A self-administered instrument of 40 items (20 for each subscale) that measures the frequency of which anxiety reactions occur and is evaluated on a 4-point Likert scale. In the state of anxiety subscale, item scores range from 0 = not at all, 1 = somewhat, 2 = moderately, and 3 = very much. On the anxiety trait subscale, the response options range from 0 = almost never, 1 = sometimes, 2 = often, and 3 = almost always. The range of scores in each subscale is from 0 to 60, with a high score meaning a worse level of anxiety [20]. The reliability of the subscales in the Spanish version were: anxiety state Cronbach’s α = 0.77; anxiety trait Cronbach’s α = 0.64 [21].

Pittsburgh Sleep Quality Questionnaire: A self-administered questionnaire consisting of 18 questions that assesses sleep quality during the last month. It includes 7 components and scores that measure sleep quality through subjective quality, sleep latency, sleep duration, regular sleep efficiency, sleep disorders, use of sleep medications, and daytime dysfunction. A total of 21 points can be obtained. The higher the score, the worse the sleep will be [22]. The internal consistency of the Spanish version has a Cronbach’s α = 0.805, the test–retest reliability is 0.773 (Pearson’s correlation) [23].

### 2.5. Procedure

A document was prepared that included the evaluation scales selected for the study. The link of this questionnaire was sent individually by email to the university’s teaching staff, through a Google Drive form, informing them about the study, its objective, the confidentiality of the data, and the requisite of informed consent. The study was carried out from May to June 2019 through self-administered questionnaires.

### 2.6. Data Analysis

For the data analysis, the statistical program SPSS version 22 was used. First, a descriptive analysis was carried out. An analysis of the distribution of the sample was carried out through the K-S test. A comparative analysis was performed between the two groups for all descriptive and outcome variables. The differences between the groups were analyzed using the Student’s t-test or Wilcoxon’s test for the parametric or non-parametric variables, respectively. To analyze eventual multicollinearity between all outcome variables, a correlation between outcome variables was performed using the Pearson correlation coefficient. Correlation levels were classified using the following field scale [24]: strong: r ≥ 0.75; moderate: 0.50 ≤ r ≤ 0.74; poor: r ≤ 0.49. In addition, through a linear regression, an estimation of the degree of contribution was calculated that each independent variable (burnout syndrome, anxiety level, depression state, and sleep quality) has on the dependent variable (TMD).

## 3. Results

The study population was divided into two groups of university teaching staff; on the one hand were university teaching staff with no contractual stability and, on the other, university teaching staff with contractual stability. Table 1 shows the total means and standard deviation, both in the total of participants and in each of the groups separately (university teaching staff with no contractual stability and university teaching staff with contractual stability) in relation to each of the outcome variables included in the study. When analyzing the descriptive variables of the sample (age, height, weight, and body mass index), significant differences were observed between both groups in all the variables analyzed with the exception of the mean height of the group (Table 1). On the other hand, when the comparison between the different groups is analyzed, it is observed how there are significant differences (*p* < 0.05) in practically all the outcome variables (state of depression, anxiety level, quality of sleep, emotional exhaustion dimension of the MBI, depersonalization dimension of the MBI, and temporomandibular dysfunction), except the personal achievement dimension of the MBI (Table 1).

Table 2 presents, quantitatively (lower part) and qualitatively (upper part), the levels of correlation between the different outcome variables analyzed in the present study. In addition, it also indicates the levels of significance in the total number of participants between each of the variables included in the study. There is a strong correlation between the level of anxiety trait and state of depression, between the level of anxiety trait and level of anxiety state and between the state of depression and level of temporomandibular dysfunction (*p* ≤ 0.01), while a poor and inversely proportional correlation exists between the quality of sleep and personal achievement (*p* ≤ 0.01). Additionally, on the other hand, there is no strong correlation between each of the dimensions of burnout and temporomandibular dysfunction. (Table 2).

On the other hand, Table 3 presents the quantitative values of the correlation levels considering each of the contractual figures (university teaching staff with no contractual stability and university teaching staff with contractual stability). The bottom part of the Table 3 indicates the correlations of staff with no contractual stability, being able to observe how the correlation levels oscillate between r = 0.876 (anxiety state and level of anxiety trait (*p* = 0.01)) and r = 0.238 (FAI and MBI Burnout Depersonalization). On the other hand, the university teaching staff with contractual stability’s results are at the top of the Table 3, observing correlation levels that oscillate between r = 0.832 (STAI State, STAI Trait) and r = 0.068 (FAI, Pittsburg). The rest of the correlation data can be observed in Table 3.

Finally, a linear regression where the temporomandibular dysfunction measured with the FAI has acted as the dependent variable, while the variables related to psychological aspects have acted as independent variables (BDI, STAI, Pittsburgh, and MBI). In the total number of participants, the independent variables manage to explain up to 62% of the results obtained on the dependent variable. However, this level of explanation presents a different picture when university teaching staff with contractual stability and university teaching staff with no contractual stability are analyzed separately. In the group of staff with no contractual stability, the psychological variables (independent variables) manage to explain over 75% of the results obtained on the dependent variable. In contrast, in the group of university teaching staff with contractual stability, the proportion of the results of the dependent variables (FAI) falls below 40% that could be explained by the psychological variables (independent variables) (Table 4).

## 4. Discussion

The objective of this study was to analyze burnout syndrome, anxiety level, state of depression, and sleep quality in the university teaching staff and its impact on temporomandibular dysfunction, as well as to analyze the possible relationship that may exist between psycho-emotional variables and the possibility of suffering from temporomandibular disorders. In the results of this study, it can be seen (Table 1) that the scores are higher in the group of university teaching staff with no contractual stability compared to university teaching staff with contractual stability in relation to the psycho-emotional variables and temporomandibular dysfunction and how the psycho-emotional variables can influence the presence or absence of temporomandibular dysfunction (Table 4) depending on job stability. Based on the observed results, it could be confirmed that the objective of the study was reached, and the starting hypothesis is confirmed.

### 4.1. Correlations between Psycho-Emotional Variables and Temporomandibular Dysfunction

This study presents a multiple regression model (Table 4), where it can be seen that, in the total number of participants, the psycho-emotional variables explain up to 62% of the obtained results in the temporomandibular dysfunction variable. However, when the analysis is carried out with the job stability of university professors in mind, a different behavior is observed in the two analyzed groups (university teaching staff with no contractual stability and university teaching staff with contractual stability). Specifically, in the group of staff with no contractual stability, the psycho-emotional variables (independent variables) explain over 75% of the results obtained on the dependent variable (FAI). However, when analyzing the group of university teaching staff with contractual stability, the level drops to below 40% the proportion of the results on the dependent variable, which could be explained by the psycho-emotional variables. No study has been found that explicitly analyzes the percentage that psychological factors can have on the probability of suffering from temporomandibular alterations. Nevertheless, studies exist that have demonstrated that psycho-emotional factors, such as anxiety or depression, could be described as possible predisposing or perpetuating factors for temporomandibular dysfunction [25,26] as well as stress [25,27]. This tendency to suffer from temporomandibular dysfunction problems may be influenced by job instability, which increases the risk of suffering from psycho-emotional changes and may have repercussions in the presence of temporomandibular dysfunction, being a set of related factors that revolve around the situation of job instability. Previous studies exists that affirm that job insecurity is a source of stress in university teaching staff with no contractual stability [28] due to the fact that workers lack contracts that guarantee the full benefits of social security, feel affected by the discontinuity of employment, are unable to plan a personal future due to job instability, and the consequences that this stress can have on their health [29]. These factors influence a poorer quality of research, reduced competition and innovation, reduction in research funds, and results in high health costs associated with stress [30]. On the other hand, the association between psychological distress and depression are linked to job instability, taking into account factors such as lower salary, worse working conditions, fewer opportunities for advancement, less generous benefits, and even less satisfactory hours [31].

Likewise, it should be noted that among staff with no contractual stability, there is a poorer quality of sleep associated with greater temporomandibular dysfunction (Table 1). These results coincide with another previous study, in which temporomandibular dysfunction, sleep disorders, stress, and their association were analyzed. It concluded that sleep quality influences the occurrence of temporomandibular dysfunction and that a high percentage had a sleep disorder and temporomandibular dysfunction [32]. This can also be explained in the results of the present study with the multiple regression analysis (Table 4) as the psycho-emotional variables (independent variables) manage to explain over 75% of the results obtained on the dependent variable (FAI), among them also taking into consideration sleep quality.

### 4.2. Psychological Disorders Associated with Job Instability

As it can be observed in Table 1, the university teaching staff with no contractual stability present psychological profiles characterized by emotional exhaustion and depersonalization, therefore a higher incidence of suffering from burnout syndrome [33]. There could be multiple reasons for this differentiation between the university teaching staff with contractual stability and staff with no contractual stability, although it could be highlighted among others, the feeling of being less compensated for their work, being obliged to assume a teaching load equal to or greater than university teaching staff with contractual stability, or the constant feeling of being considered for job promotion [34].

Temporality in the workplace is associated with suffering from burnout in other professions such as in the healthcare field, where personnel who are temporarily hired are those most affected by burnout syndrome [35]. This stress may be associated with the anxiety that workers suffer from when finishing each contract due to job uncertainty and having to face financial and sustainability commitments of a socio-personal nature [36].

In any case, the results show that job insecurity could be one the main reasons for feeling burned out, with higher levels of anxiety and depression among university teaching staff with no contractual stability also being notable (Table 1). This may negatively affect the mental health and psycho-affective sphere of teachers, therefore reducing the ability to perform their job functions due to a higher prevalence of symptoms such as mental fatigue, stress, anxiety, forgetfulness, frustration, nervousness, anguish, insomnia, and depression, as reported in previous studies [37]. At the same time, the results can be compared to other professions, such as in the case of healthcare personnel, where a strong association between depression and burnout syndrome can be noted [38]. Additionally, the data collected in the present study on higher levels of depersonalization and emotional exhaustion in university teaching staff with no contractual stability (Table 1) can be contrasted with other previous studies in which high scores of depersonalization in teachers correspond to high scores in emotional exhaustion [39,40]. Furthermore, the extent of burnout syndrome in university professors is comparable to that of other areas of teaching and health professions [40].

It is important to bear in mind that the submission of the request for participation was made in a blinded and independent manner; however, perhaps the profile of the teaching university staff that responded to the different questionnaires could have a tendency to suffer psychological alterations. This fact could be considered a weakness of the study that should be taken into account when interpreting the results presented in this study.

## 5. Conclusions

Based on the results of this study, it can be said that university teaching staff with no contractual stability demonstrate higher levels of depression, anxiety, emotional exhaustion, depersonalization, and poorer sleep quality in comparison to university teaching staff with contractual stability. Furthermore, these variables manage to explain up to a 77.8% probability that university teaching staff with no contractual stability suffer from temporomandibular dysfunction, in comparison to 44.2% of university teaching staff with contractual stability.

## Figures and Tables

**Table 1:** Average and standard deviation of university teaching staff with no contractual stability and university teaching staff with contractual stability.

	Total Participants	Staff with No Contractual Stability	University Teaching Staff with Contractual Stability	*p*-Value (Between Both Groups)
Age (years)	46.78 (±8.98)	41.34 (±9.36)	51.15 (±5.72)	0.001
Height (cm)	168 (±6.49)	172 (±7.88)	166 (±7.15)	0.003
Weight (Kg)	72.5 (±8.98)	71.21 (±6.01)	73.09 (±5.19)	0.179
BMI	25.81 (±3.18)	24.37 (±3.29)	26.53 (±4.59)	0.038
BDI	5.24 (±6.29)	6.42 (±7.34)	4.30 (±5.14)	0.028
STAI Status	34.64 (±12.08)	37.87 (±13.56)	32.05 (±10.09)	0.001
STAI Trait	29.85 (±10.30)	32.55 (±11.60)	27.69 (±8.58)	0.002
Pittsburgh	5.10 (±3.01)	5.66 (±3.20)	4.65 (±2.79)	0.027
MBI Burnout—Emotional exhaustion	18.97 (±7.36)	21.22 (±8.57)	17.17 (±5.65)	0.001
MBI Burnout—Depersonalization	8.43 (±3.37)	9.45 (±3.78)	7.61 (±2.75)	0.001
MBI Burnout—Personal achievement	31.78 (±5.38)	31.97 (±5.73)	31.63 (±5.11)	0.673
FAI	16.12 (±11.14)	18.30 (±13.12)	14.36 (±8.93)	0.020
N (females/males)	173 (81/92)	77 (41/36)	96 (40/56)	

BDI: Beck Depression Inventory; FAI: Fonseca Anamnestic Index; MBI: Maslach Burnout Inventory; STAI: State and Trait Anxiety Questionnaire.

**Table 2:** Pearson’s correlations in the total number of participants.

	BDI	STAI State	STAI Trait	Pittsburgh	MBI Burnout Emotional Exhaustion	MBI Burnout Depersonalization	MBI Burnout Personal Achievement	FAI
BDI	1	Moderate	Strong	Poor	Moderate	Poor	Poor	Strong
STAI State	0.724(**)	1	Strong	Poor	Moderate	Moderate	Moderate	Moderate
STAI Trait	0.771(**)	0.866(**)	1	Poor	Moderate	Moderate	Moderate	Moderate
Pittsburgh	0.480(**)	0.464(**)	0.454(**)	1	Poor	Poor	Poor	Poor
MBI Burnout Emotional exhaustion	0.615(**)	0.721(**)	0.715(**)	0.355(**)	1	Moderate	Poor	Moderate
MBI Burnout Depersonalization	0.396(**)	0.504(**)	0.509(**)	0.289(**)	0.551(**)	1	Poor	Poor
MBI Burnout Personal achievement	−0.470(**)	−0.517(**)	−0.530(**)	−0.283(**)	−0.434(**)	−0.460(**)	1	Poor
FAI	0.789(**)	0.609(**)	0.626(**)	0.404(**)	0.504(**)	0.304(**)	−0.301(**)	1

BDI: Beck Depression Inventory; FAI: Fonseca Anamnestic Index; MBI: Maslach Burnout Inventory; STAI: State and Trait Anxiety Questionnaire; * *p* ≤ 0.05, ** *p* ≤ 0.01.

**Table 3:** Pearson’s correlations in different contractual figures.

	BDI	STAI State	STAI Trait	Pittsburgh	MBI Burnout Emotional Exhaustion	MBI Burnout Depersonalization	MBI Burnout Personal Achievement	FAI
BDI	1	0.582 (**)	0.681 (**)	0.232 (*)	0.493 (**)	0.407 (**)	−0.404 (**)	0.632 (**)
STAI State	0.802 (**)	1	0.832 (**)	0.269 (**)	0.627 (**)	0.392 (**)	−0.459 (**)	0.410 (**)
STAI Trait	0.818 (**)	0.876 (**)	1	0.248 (*)	0.656 (**)	0.396 (**)	−0.477 (**)	0.483 (**)
Pittsburgh	0.647 (**)	0.586 (**)	0.583 (**)	1	0.161	0.210 (*)	−0.195	0.068
MBI Burnout Emotional exhaustion	0.662 (**)	0.749 (**)	0.721 (**)	0.449 (**)	1	0.483 (**)	−0.398 (**)	0.361 (**)
MBI Burnout Depersonalization	0.345 (**)	0.523 (**)	0.528 (**)	0.295 (**)	0.535 (**)	1	−0.467 (**)	0.324 (**)
MBI Burnout	−0.550 (**)	−0.612 (**)	−0.622 (**)	−0.387 (**)	−0.516 (**)	−0.509 (**)	1	−0.162
Personal achievement								
FAI	0.872 (**)	0.709 (**)	0.693 (**)	0.626 (**)	0.550 (**)	0.238 (*)	−0.430 (**)	1

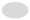
 Staff with no contractual stability; 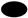 Staff with contractual stability; * *p* ≤ 0.05 ** *p* ≤ 0.01.

**Table 4:** Linear regression.

Model	Total Group		University Teaching Staff with No Contractual Stability		University Teaching Staff with Contractual Stability	
R	0.797 (a)		0.882 (a)		0.665 (a)	
R^2^	0.635		0.778		0.442	
R^2^ Adjusted	0.620		0.756		0.398	
	Stand.Coeff	T (Sig.)	Stand.Coeff	T (Sig.)	Stand.Coeff	T (Sig.)
(Constant)		−0.490 (0.625)		0.391 (0.697)		−0.888 (0.377)
BDI	0.747	90.628 (0.000)	0.823	70.172 (0.000)	0.569	50.104 (0.000)
Anxiety_state	0.115	10.140 (0.256)	0.153	10.152 (0.253)	0.029	0.198 (0.843)
Anxiety_trait	−0.001	−0.011 (0.991)	−0.082	−0.609 (0.545)	0.130	0.780 (0.437)
Pittsbrurg_sleep	0.024	0.446 (0.656)	0.108	10.438 (0.155)	−0.096	−10.146 (0.255)
MBI_BET	0.009	0.121 (0.904)	−0.034	−0.379 (0.706)	0.002	0.015 (0.988)
MBI_BD	−0.007	−0.122 (0.903)	−0.069	−0.925 (0.358)	0.137	10.391 (0.168)
MBI_BPR	0.116	20.005 (0.047)	0.054	0.708 (0.481)	0.189	10.948 (0.055)

(a) Dependent Variable: FAI: Fonseca Anamnestic Index; MBI BD: MBI Burnout Depersonalization; MBI_BET: MBI Burnout Emotional Tiredness; MBI BPR: MBI Burnout Personal Realization.
